# Supplementary material for: Oral Immunization with Attenuated Salmonella Choleraesuis Expressing the FedF Antigens Protects Mice against the Shiga-Toxin-Producing Escherichia coli Challenge
Source: Biomolecules. 2023 Nov 30;13(12):1726. doi: 10.3390/biom13121726 (PMC10741478; doi:10.3390/biom13121726)
Supplement: Supplementary file 1 [file biomolecules-13-01726-s001.zip › biomolecules-2699606-supplementary.pdf]

Supplementary figure

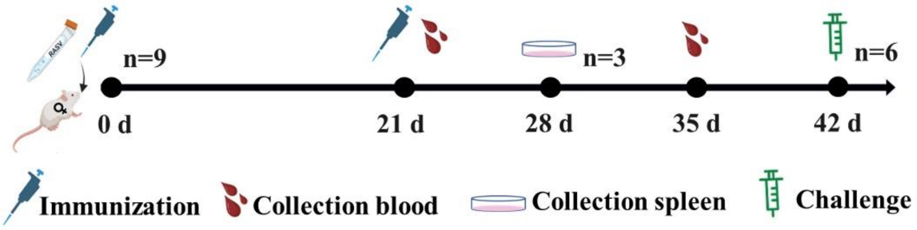

Figure S1. Schematic diagram of the immunization and challenge experiment.

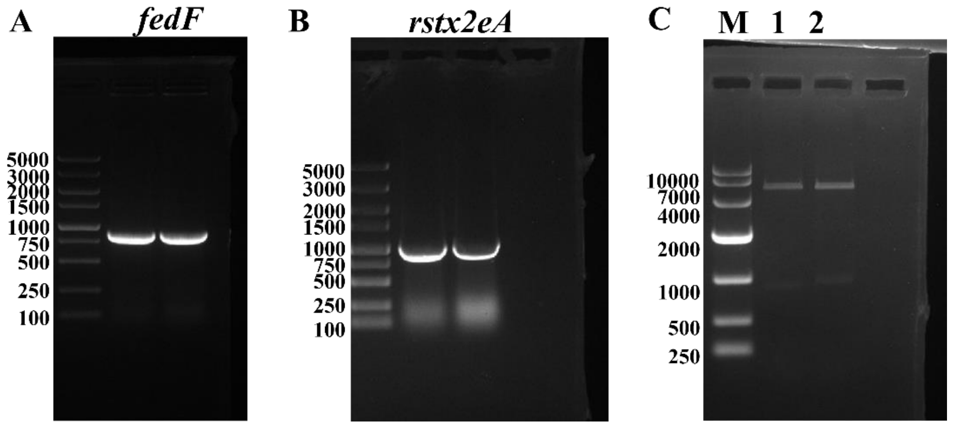

Figure S2. Original images of figure 1A-C.

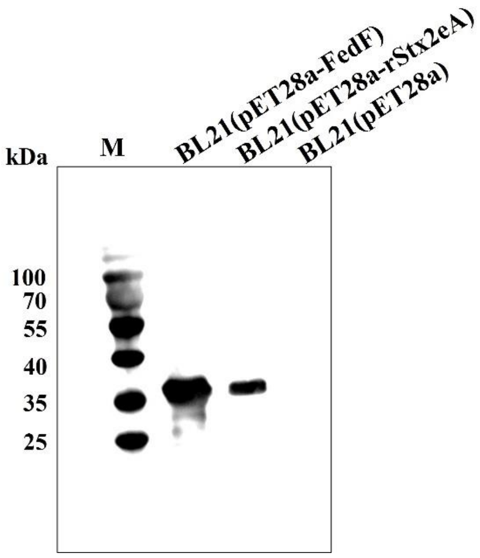

Figure S3. Original images of figure 1D.

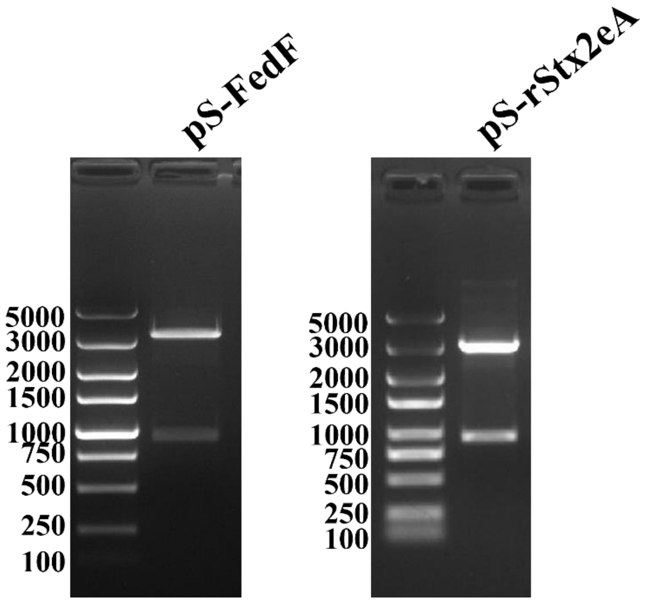

Figure S4. Original images of figure 2B.

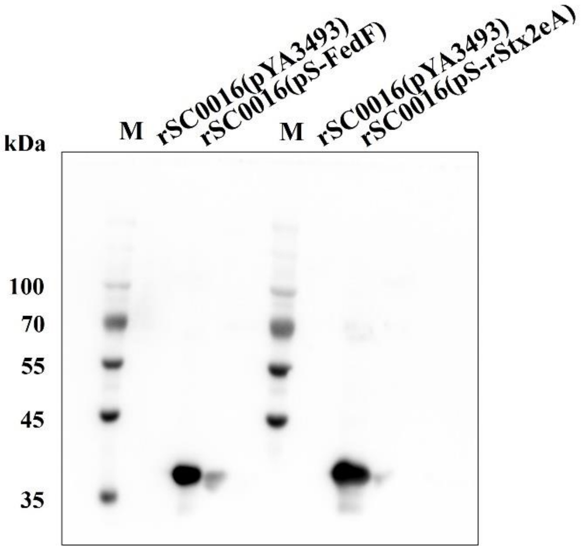

Figure S5. Original images of figure 2C.

Supplementary table

Table S1 Median lethal dose results of strain STEC20.

Group	Dose	Number of animals	Number of deaths	Mortality rate	LD50
1	3.2×10^9	5	5	100%	5.62×10^7
2	3.2×10^8	5	4	80%	
3	3.2×10^7	5	2	40%	
4	3.2×10^6	5	1	20%	
